# The Complexity of Antibody-Dependent Enhancement of Dengue Virus Infection

**DOI:** 10.3390/v2122649

**Published:** 2010-12-08

**Authors:** Maria G. Guzman, Susana Vazquez

**Affiliations:** Department of Virology, PAHO/WHO Collaborating Center for the Study of Dengue and its Vector, “Pedro Kouri” Tropical Medicine Institute of Havana, Cuba; E-Mail: ciipk@ipk.sld.cu

**Keywords:** dengue, dengue hemorrhagic fever, prM, ADE, neutralization, cleavage

## Abstract

Antibody-dependent enhancement (ADE) has been proposed as a mechanism to explain dengue hemorrhagic fever (DHF) in the course of a secondary dengue infection. Very recently, Dejnirattisai *et al.*, 2010 [1], published an important article supporting the involvement of anti-prM antibodies in the ADE phenomenon. The complexity of ADE in the context of a secondary dengue infection is discussed here.

## 1. Introduction

A rapid increase in dengue reports has been observed in the last three decades. Today, dengue infections are a serious cause of morbidity and mortality in most tropical and subtropical regions of the world: an estimated 50–100 million people are infected annually and over 2.5 billion people live in endemic areas; and more than 100 countries are at risk for dengue transmission. The disease is endemic in Asia, the Pacific, the Americas, Africa and the Middle East [2,3].

Dengue is caused by four antigenically related viruses (DENV 1–4) within the family *Flaviviridae*, genus *Flavivirus*. These are transmitted to humans by *Aedes* mosquito bites, and *Aedes aegypti* is the main vector. The genome of these enveloped single-strand positive-polarity RNA viruses codes for three structural proteins (capsid C, membrane, M, and the envelope, E) and seven non-structural proteins (NS1, NS2A, NS2B, NS3, NS4A, NS4B, NS5) [4].

Two types of virions are recognized: mature extracellular virions contain M protein, while immature intracellular virions contain prM, which is proteolytically cleaved during maturation to yield M protein.

The envelope of the virus contains the viral surface proteins E and M. The E glycoprotein has important functional roles in virus attachment to cells and fusion with membranes, and is the major target for neutralizing antibody. It contains the main epitopes recognized by neutralizing antibodies (virus-specific and cross-reactive epitopes) [5,6]. This protein has three structural and functional domains: domain II contains the internal fusion peptide (responsible for the fusion of flaviviruses to their target cells) and domain III the cellular receptor-binding motifs [7,8]. Domains I and III contain predominantly subcomplex- and type-specific epitopes, whereas domain II contains the major flavivirus group and subgroup cross-reactive epitopes [9–11].

M protein may be found in two forms. In cell-associated (immature) virions, prM (the precursor of M protein) is observed, which forms a heterodimer with the E protein (prM-E heterodimer). Apparently, prM serves as a chaperone for the E protein, protecting it from irreversible inactivation during transport of virions to the cell surface in acidic post-Golgi vesicles [12,13]. Through this association, prM participates in the viral assembly and budding into the lumen of the endoplasmic reticulum. Intracellular virions remain non-infectious until release when they are converted to infectious form through the cleavage of prM into the soluble pr peptide and the particle associated M protein by a host-cell-derived furin-like protease [14].

Uncleaved prM prevents the E protein from undergoing the structural changes that are required for low-pH-induced membrane fusion of DENV. Therefore, fully immature DENV is essentially non-infectious [15]. Depending on the extent of prM cleavage, the extracellular particles may contain varying proportions of prM and M. Levels of around 30% of prM containing immature particles have been reported in DENV infected cells [16, 17]. The charged residues surrounding the furin consensus sequence at the prM cleavage junction could partially explain lower or higher cleavage efficiency; in addition, structural differences inherent to flaviviruses at prM junction affect prM cleavability [18].

## 2. Dengue Hemorrhagic Fever, Secondary Infection and Antibody-Dependent Enhancement

Dengue infection can be asymptomatic or present in two clinical forms of illness, dengue fever (DF) and the more severe dengue hemorrhagic fever/dengue shock syndrome (DHF/DSS). Plasma leakage, hemorrhage and thrombocytopenia characterize DHF/DSS [19,20].

Single-serotype natural infections result in lifelong immunity to the infecting serotype but only short-term cross-protection against heterotypic serotypes [21]. The humoral response to dengue infection is important for controlling infection and virus dissemination. Despite antigenic relatedness of viruses in the dengue complex, two or more serotypes may sequentially infect one individual. Specific neutralizing IgG antibody against the infecting DENV lasts decades, while cross-reactive neutralizing activity declines over time [22,23]. Preliminary reports also suggest that in human beings there is a continuous selection process of populations of dengue-virus neutralizing-antibodies with increasing homologous reactivity and concurrent decrease in heterotypic cross reactions [24].

Early studies in Thailand recognized that DHF/DSS peaked in two populations: first-time infected infants born to dengue-immune mothers and children who had experienced a mild or asymptomatic dengue infection and become secondarily infected by a different dengue serotype [25,26]. These studies suggested that DHF/DSS is 15–80 times more frequent in secondary infections than in primary ones, and that up to 99% of DHF cases reveal heterotypic antibodies to the dengue serotype causing the DHF [27].

These first observations were confirmed in a different setting. The DENV 2 epidemic of 1981 (preceded by a mild epidemic of DENV 1 in 1977) reported in Cuba, supported secondary infection as a main risk factor for the severe forms of dengue infection. In this epidemic of more than 300,000 cases, 10,000 severe and very severe cases and 158 fatalities (101 children), secondary infection in the sequence DENV 1/DENV 2 was demonstrated in 98% of the DHF/DSS cases [28–30]. In addition, DHF/DSS did not occur in children of 1–2 years. They were born after the 1977 epidemic and, consequently, in 1981, they were at risk only of primary DENV infection [29]. More than 20 years after the DENV 1 epidemic, secondary infection as a main risk factor for DHF/DSS was confirmed again in the Cuban epidemics of 1997 (DENV 2) and 2001–02 (DENV 3) [31–35].

To explain the association of secondary infection to severe illness, Antibody-Dependent Enhancement (ADE) was proposed as the immune system’s mechanism to enhance viral pathogenesis. ADE has been described for several viruses including DENVs, measured by *in vitro* enhancement of cell infection [36–38]. Also, monkeys passively immunized concurrently with a DENV infection developed a higher viremia than control animals [39]. More recently, Goncalvez *et al.* [40] demonstrated a significant increase of DENV 4 viremia titers in monkeys passively immunized with transferred dilutions of an anti-dengue humanized monoclonal antibody [40].

In humans, indirect evidence of ADE has been reported. ADE was observed *in vitro* in sera from mothers whose infants developed DHF after a primary dengue infection [41]. This study demonstrated that maternal antibody to DENV declines at a constant rate and passes in time through three functional states: neutralization, enhancing virus growth and antibody degradation. This early study suggested that as anti-dengue antibody to a first infection wanes, some individuals will experience an interval during which their antibody level will drop below its protective capacity, acquiring the power to enhance infection. In another study, Kliks *et al.* [42] reported that undiluted pre-infection sera from children who developed DHF were more likely to show enhancement of the dengue virus infection than pre-infection sera of children with an asymptomatic secondary infection.

Evidence suggests that enhancing and cross-reactive neutralizing antibodies regulate dengue epidemics and disease severity. In this sense, epidemiological and serological observations made during the Cuban dengue epidemics support the role of secondary infection and ADE even 20 or more years after primary dengue infection. A marked increase in severity associated with the longer of the two intervals (20 years *versus* four years) between an initial DENV1 infection and a secondary DENV 2 (Asian genotype) infection has been reported [43]. In addition, some sequences of infection such as DENV 1 followed by DENV 2, and DENV 1 followed by DENV 3 have been associated with greater disease severity [44,45].

## 3. The Antibody-Dependent Enhancement Phenomenon

ADE occurs when antibody-virus complexes are internalized into cells via FcγRs resulting in infection of a higher number of target cells, which may lead to higher viral production. Cross-reactive antibodies lacking neutralizing activity are induced during a primary dengue infection. In secondary infection, these antibodies bind to the second infecting virus. Increased viral production has typically been interpreted to be the result of an increased number of infected Fcγ-R-bearing cells and possibly the result of an accelerated rate of internalization and cell infection by immune-complexes [46–48]. A higher viremia in infected patients and consequent greater severity has been hypothesized [49]. Studies reported by Vaughn *et al.* and Libraty *et al.* observed a higher viremia and NS1 antigenemia in children with DHF than in those with DF [50,51]. Also in Taiwanese patients it was observed that dengue RNA titers even after defervescence, correlated with disease severity [52]. In a more recent study, Cameron *et al.* reported heterogeneity in viremia and NS1 antigenemia in Vietnamese infants with DHF in the course of their primary DENV infection; however these determinations were made at the time of hospital admission. They also found that these infants experienced DHF when the maternally-derived neutralizing antibody titer had declined to <1:20 [53].

A complementary mechanism to higher viremia explaining disease severity as a consequence of ADE may be that FcγR-mediated entry suppresses the antiviral immune response. An *in vitro* study with Ross River virus showed that viral entry via the FcR pathway could suppress antiviral genes and enhance IL-10 production, while entry via the normal mechanism does not change the antiviral environment [54]. In the case of dengue, it has been reported that infection of THP-1 cells via FcR also suppresses transcription and production of IL-12, IFNγ, TNFα and NO, but enhances the expression of anti-inflammatory cytokines [55] with a milieu change favorable to viral replication. These observations suggest that ADE of DENV infection not only facilitates the virus entry process but also could modify innate and adaptive intracellular antiviral mechanisms [55].

Studies using monoclonal antibodies have demonstrated that enhancing antibodies are directed to E and prM proteins [56]. Although both proteins seem to be involved in neutralization and the ADE mechanism, more studies have been designed to evaluate the role of E protein. Greater understanding of the antibody-neutralization mechanisms could shed light on their likelihood of promoting ADE.

## 4. The Neutralization Mechanism

Flavivirus neutralization is a multiple-hit phenomenon requiring engagement by more than one antibody. Neutralization occurs when the number of antibodies bound to an individual virion exceeds a required threshold, antibody affinity and accessibility of epitopes on virus particles playing an important role [57]. Neutralizing antibodies directed mostly to E protein inhibit viral attachment, internalization and/or replication within the cell [58]. These E-specific antibodies appear to be pivotal, mediating homologous protection against dengue reinfection; however, in mice, prM vaccine has also been shown to protect against the lethal DENV challenge [59].

Neutralization at low occupancy requires lower antibody concentrations and can occur with lower-affinity antibodies, while those antibodies specific to poorly accessible epitopes require relatively high concentrations. Most epitopes have the capacity to elicit antibodies capable of promoting ADE [60]; however, antibodies specific to poorly accessible epitopes are more likely to promote ADE over a wide range of concentrations [61,62]. Recently, Lok *et al.* [63] showed that a partially occluded epitope may become available to antibody binding under certain conditions, suggesting that the virus is in dynamic motion making hidden epitopes briefly available [63].

Despite the large body of work with mouse monoclonal antibodies, little has been done to characterize the binding properties of human dengue immune sera and to understand the relationship between human antibody binding, neutralization and ADE [64]. Recent studies have proposed that the major cross-reactive and serotype-specific neutralizing epitopes targeted by human immune sera are inter-domain epitopes and/or located outside of domain III of E protein [40,65]. Wahala *et al.* observed that, unexpectedly, domain III-binding antibodies play a minor role in DENV neutralization. In addition, in another report these authors suggest that type-specific epitopes on domain III are not conserved between strains of DENV3 [66]. Previous investigations support large differences in neutralization titers when comparing different genotypes of the same virus [67,68].

## 5. Potential Role of Immature DENV in Antibody-Dependent Enhancement

Recently, Dejnirattisai *et al.* [1] generated a panel of human monoclonal antibodies to DENV. They observed that (a) antibodies to prM were a major component of the response, highly cross-reactive among the dengue serotypes, and (b) these antibodies have potent ADE activity and low neutralization capacity. Considering these results, the authors propose that partial cleavage of prM reduces antigen density availability for viral neutralization, leaving the viruses susceptible to ADE by antibody to prM.

Previously, a host-protective effect of anti-prM was reported for DENV; however, how these antibodies would exert their effect was not clear [18,59,69–71]. It has been proposed that weak neutralization of dengue infectivity by some anti prM monoclonal antibodies and anti-prM peptide sera could be due to their cross-reactivity with E protein [18,70,71]. However, similar levels of enhancing activity by strongly enhancing anti-E monoclonal antibodies have been previously reported [56,72]. Some studies report enhancement of infection presumably due to the presence of uncleaved prM in virus preparations, but also with DENV particles containing high levels of prM after cell treatment with chloroquine [72,73]. Apparently, infection enhancement and lack of potent neutralization are common properties of anti-prM antibodies, suggesting that prM constitutes another target for infection-enhancing antibodies but also that extracellular dengue virions containing prM could be infectious [18].

Previous studies have shown that immature particles are non-infectious, since the presence of prM obstructs the low-pH-induced conformational changes in the E protein required for membrane fusion of the virus [15,74]. However, very recently, Rodenhuis-Zybert *et al.* [75] showed that fully immature dengue particles become highly infectious when interacting with prM antibodies. They showed that lack of infectivity of immature particles in the absence of antibodies was related to inefficient binding of immature virions to cell surfaces, but if binding is facilitated through anti-prM antibodies, immature particles become highly infectious, presumably due to efficient intracellular processing of prM to M by furin activity within the target cell. These antibodies facilitate efficient binding and cell entry by immature particles into Fc-R- expressing cells [75].

Together, these observations suggest that immature viral particles have the potential to be highly infectious and hence may contribute to development of the severe disease during secondary infection [75]. Consequently, it is important to define the possible *in vivo* effects of maintaining prM on the virion surface but also the viral and host factors involved in the efficiency of prM cleavage. It has been suggested that alteration of furin target sequence in the prM junction can affect virus export [76]. Also, several studies have suggested that the multiplication of flaviviruses is not self-reliant and that the viruses subvert cellular proteins to become part of their replication strategy [77,78].

Of interest is that Dejnirattisai *et al.* [1] found that antibodies to prM were a major component of the anti-dengue response. Previous reports recognized that the main response is directed to E protein, but also support that anti-prM antibodies are generated during dengue infection in humans [79–81]. It cannot be excluded that these apparently dissimilar observations depend on the characteristics of the tested samples and the employed methodologies.

CryoEM images have shown that WNV and DENV preparations contain a mixture of immature, partially mature and mature viral particles, most likely due to incomplete processing by furin during maturation [82]. Cherrier *et al.* showed that an epitope within the fusion loop of WNV E protein is largely inaccessible in mature virions but that a cross-reactive fusion-loop antibody with low neutralization activity binds preferentially to the spikes in immature virions [82]. In a secondary infection, these antibodies may promote infection through ADE by augmenting attachment and/or entry of partially immature virions. The fact that an antibody neutralizes infectivity by binding to an immature virion supports the hypothesis that hybrid mature/immature particles can contribute to virus infectivity and pathogenesis [82]. These observations can also be extended to anti prM antibody.

**Figure 1:**
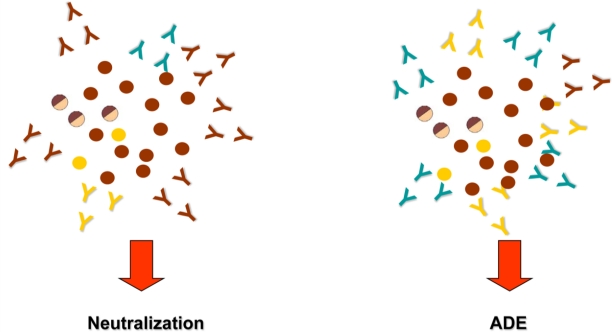
Schematic representation of viral populations and anti-E and anti-prM antibodies involved in neutralization and ADE mechanism. Immature ( 
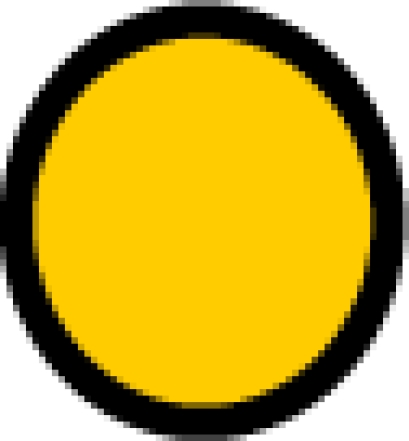
), partially mature ( 
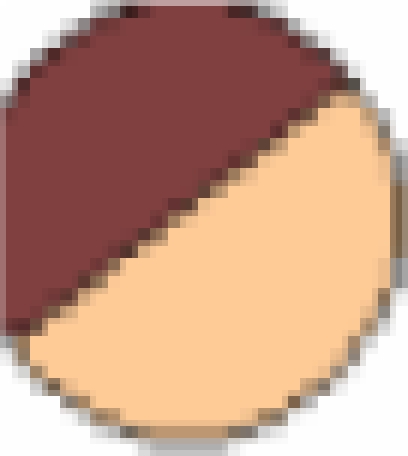
), mature ( 
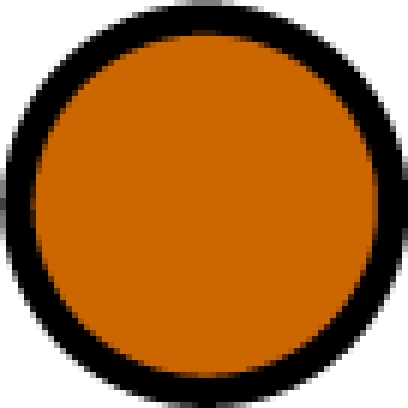
) viral particles, neutralizing anti-E antibodies ( 
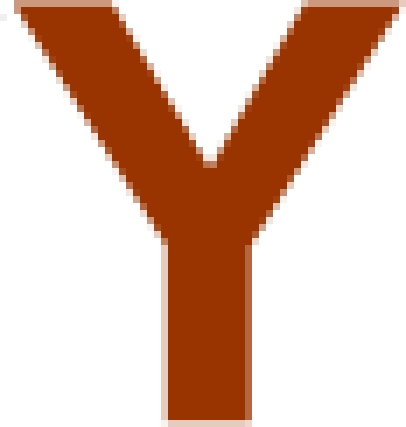
), cross-reactive non-neutralizing anti-E antibodies ( 
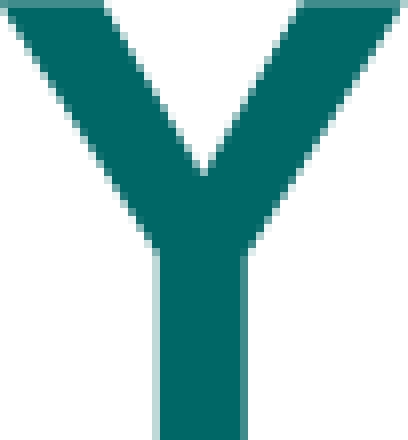
) and cross reactive anti-prM antibodies ( 
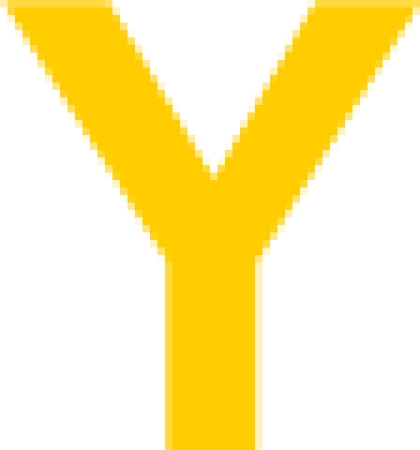
).

Figure 1 shows viral populations and anti-E and -prM antibodies involved in neutralization and ADE of DENV infection.

## 6. Conclusions

DHF/DSS is the result of the interaction of several factors in which the viral and host characteristics are important [44,83]. Some DENV genotypes have the potential to produce DHF [84]. In addition, host factors are of importance: these include age (children at higher risk than adults), ethnicity (white people at higher risk than black people), chronic diseases (bronchial asthma, diabetes mellitus, sickle cell anemia), nutritional status, sex and the individual’s genetic composition (allelic variants of genes that encode cellular receptors such as DC-SIGN and FcγRIIA, Vitamin D receptor as well as molecules involved in the antigen recognition, HLA, and cytokines have also been associated with higher or lower risk of DHF) [85–91]. However, secondary infection is considered the main risk factor for disease severity.

The dengue antibody somehow modulates subsequent infection with an enhancing or neutralizing role that up- or down-regulates dengue infection of mononuclear phagocytes (Figure 1) [92]. Consistent with current evidence and considering ADE as a central mechanism, a working hypothesis of dengue pathogenesis suggests that DHF/DSS during a secondary infection is the result of antibody-enhanced infection of mononuclear phagocytes. Immune complex infection suppresses cellular immune responses, increasing intracellular infection and generating inflammatory cytokines and chemokines that together contribute to the development of severe disease [48].

Concern over ADE and its role in DHF/DSS suggest the necessity of a tetravalent dengue vaccine stimulating a balanced and long-lasting immune response to the four serotypes. The elegant work published by Dejnirattisai *et al.* [1] adds new information to our knowledge about ADE, calling attention to the complexity of this phenomenon [1]. More research is needed to elucidate ADE’s molecular mechanisms, particularly factors influencing the final outcome of the interaction among the virus, antibody and permissive cells. Among the issues meriting careful study are the interaction of anti-prM and -E antibodies with the infecting virus to neutralize or enhance infection, the factors determining the ratio of immature/mature virion particles, the influence of ADE complement levels, and the interaction with Fc-receptors [93–96].

## References

[1] Dejnirattisai W, Jumnainsong A, Onsirisakul N, Fitton P, Vasanawathana S, Limpitikul W, Puttikhunt C, Edwards C, Duangchinda T, Supasa S (2010). Cross-reacting antibodies enhance dengue virus infection in humans. Science.

[2] Guzman MG, Kouri G (2002). Dengue: An update. Lancet Infect Dis.

[3] Gubler DJ (2002). Epidemic dengue/dengue hemorrhagic fever as a public health, social and economic problem in the 21st century. Trends Microbiol.

[4] Beasley DWC, Barrett ADT, Pasvol G, Hoffman SL (2008). The Infectious Agent. Dengue.

[5] Guirakhoo F, Heinz FX, Mandl CW, Holzmann H, Kunz C (1991). Fusion activity of flaviviruses: Comparison of mature and immature (prM-containing) tick-borne encephalitis virions. J Gen Virol.

[6] Putnak R, Feighny R, Burrous J, Cochran M, Hackett C, Smith G, Hoke C (1991). Dengue-1 virus envelope glycoprotein gene expressed in recombinant baculovirus elicits virus-neutralizing antibody in mice and protects them from virus challenge. Am J Trop Med Hyg.

[7] Crill WD, Roehrig JT (2001). Monoclonal antibodies that bind to domain III of dengue virus E glycoprotein are the most efficient blockers of virus adsorption to Vero cells. J Virol.

[8] Modis Y, Ogata S, Clements D, Harrison SC (2003). A ligand-binding pocket in the dengue virus envelope glycoprotein. Proc Natl Acad Sci U S A.

[9] Mandl CW, Guirakhoo F, Holzmann H, Heinz FX, Kunz C (1989). Antigenic structure of the flavivirus envelope protein E at the molecular level, using tick-borne encephalitis virus as a model. J Virol.

[10] Rey FA, Heinz FX, Mandl C, Kunz C, Harrison SC (1995). The envelope glycoprotein from tick-borne encephalitis virus at 2 A resolution. Nature.

[11] Roehrig JT, Bolin RA, Kelly RG (1998). Monoclonal antibody mapping of the envelope glycoprotein of the dengue 2 virus, Jamaica. Virology.

[12] Guirakhoo F, Bolin RA, Roehrig JT (1992). The Murray Valley encephalitis virus prM protein confers acid resistance to virus particles and alters the expression of epitopes within the R2 domain of E glycoprotein. Virology.

[13] Lindenbach BD, Rice CM, Knippe DM, Howley PM (2001). *Flaviviridae*: The Viruses and Their Replication. Fields Virology.

[14] Thomas G (2002). Furin at the cutting edge: From protein traffic to embryogenesis and disease. Nat Rev Mol Cell Biol.

[15] Zybert IA, van der Ende-Metselaar H, Wilschut J, Smit JM (2008). Functional importance of dengue virus maturation: Infectious properties of immature virions. J Gen Virol.

[16] Elshuber S, Mandl CW (2005). Resuscitating mutations in a furin cleavage-deficient mutant of the flavivirus tick-borne encephalitis virus. J Virol.

[17] Heinz FX, Stiasny K, Puschner Auer G, Holzmann H, Allison SL, Mandl CW, Kunz C (1994). Structural changes and functional control of the tick-borne encephalitis virus glycoprotein E by the heterodimeric association with protein prM. Virology.

[18] Sittisombut N, Keelapang P, Malasit P, Kalitzky M, Borowski P (2006). Functional role of prM glycoprotein in dengue virus replication. Molecular Biology of the Flavivirus.

[19] WHO (1997). Clinical Diagnosis. Dengue Hemorrhagic Fever Diagnosis, treatment, prevention and control.

[20] WHO (1999). Strengthening implementation of the global strategy for dengue/dengue hemorrhagic fever prevention and control.

[21] Sabin AB (1952). Research on dengue during World War II. Am J Trop Med Hyg.

[22] Halstead SB (1974). Etiologies of the experimental dengues of Siler and Simmons. Am J Trop Med Hyg.

[23] Vaughn DW, Scherer JM, Sun W, Pasvol G, Hoffman SL (2008). Resistance to infection. Dengue.

[24] Guzman MG, Alvarez M, Rodriguez-Roche R, Bernardo L, Montes T, Vazquez S, Morier L, Alvarez A, Gould EA, Kouri G (2007). Neutralizing antibodies after infection with dengue 1 virus. Emerg Infect Dis.

[25] Halstead SB (1970). Observations related to pathogensis of dengue hemorrhagic fever. VI. Hypotheses and discussion. Yale J Biol Med.

[26] Halstead SB, Nimmannitya S, Cohen SN (1970). Observations related to pathogenesis of dengue hemorrhagic fever. IV. Relation of disease severity to antibody response and virus recovered. Yale J Biol Med.

[27] Halstead SB (1982). Immune enhancement of viral infection. Prog Allergy.

[28] Kouri GP, Guzman MG, Bravo JR, Triana C (1989). Dengue haemorrhagic fever/dengue shock syndrome: Lessons from the Cuban epidemic, 1981. Bull World Health Organ.

[29] Guzman MG, Kouri G, Martinez E, Bravo J, Riveron R, Soler M, Vazquez S, Morier L (1987). Clinical and serologic study of Cuban children with dengue hemorrhagic fever/dengue shock syndrome (DHF/DSS). Bull Pan Am Health Organ.

[30] Diaz A, Kouri G, Guzman MG, Lobaina L, Bravo J, Ruiz A, Ramos A, Martinez R (1988). Description of the clinical picture of dengue hemorrhagic fever/dengue shock syndrome (DHF/DSS) in adults. Bull Pan Am Health Organ.

[31] Guzman MG, Kouri G, Valdes L, Bravo J, Alvarez M, Vazquez S, Delgado I, Halstead SB (2000). Epidemiologic studies on Dengue in Santiago de Cuba, 1997. Am J Epidemiol.

[32] Valdes L, Guzman MG, Kouri G, Delgado J, Carbonell I, Cabrera MV, Rosario D, Vazquez S (1999). [Epidemiology of dengue and hemorrhagic dengue in Santiago, Cuba 1997]. Rev Panam Salud Publica.

[33] Kouri G, Guzman MG, Valdes L, Carbonel I, del Rosario D, Vazquez S, Laferte J, Delgado J, Cabrera MV (1998). Reemergence of dengue in Cuba: A 1997 epidemic in Santiago de Cuba. Emerg Infect Dis.

[34] Pelaez O, Guzmán MG, Kourí G, Pérez R, Martín JLS, Vázquez S, Rosario D, Mora R, Quintana I, Bisset J (2004). Dengue 3 epidemic in Havana, 2001. Emerg Infect Dis.

[35] Gonzalez D, Castro O, Perez J, Martinez E, Vazquez S, Rosario D, Cancio R, Guzman MG (2005). Classical dengue hemorrhagic fever resulting from two dengue infections spaced 20 years or more apart: Havana, Dengue 3 epidemic, 2001–2002. Int J Infect Dis.

[36] Ghiasi H, Perng GC, Nesburn A, Wechsler S (2000). Antibody-dependent enhancement of HSV-1 infection by anti-gK sera. Virus Res.

[37] Girn J, Kavoosi M, Chantler J (2002). Enhancement of coxsackievirus B3 infection by antibody to a different coxsackievirus strain. J Gen Virol.

[38] Hober D, Chehadeh W, Bouzidi A, Wattré P (2001). Antibody-dependent enhancement of coxsackievirus B4 infectivity of human peripheral blood mononuclear cells results in increased interferon-alpha synthesis. J Infect Dis.

[39] Halstead SB (1979). *In vivo* enhancement of dengue virus infection in rhesus monkeys by passively transferred antibody. J Infect Dis.

[40] Goncalvez AP, Engle RE, St Claire M, Purcell RH, Lai CJ (2007). Monoclonal antibody-mediated enhancement of dengue virus infection *in vitro* and *in vivo* and strategies for prevention. Proc Natl Acad Sci U S A.

[41] Kliks SC, Nimmanitya S, Nisalak A, Burke DS (1988). Evidence that maternal dengue antibodies are important in the development of dengue hemorrhagic fever in infants. Am J Trop Med Hyg.

[42] Kliks SC, Nisalak A, Brandt WE, Wahl L, Burke DS (1989). Antibody-dependent enhancement of dengue virus growth in human monocytes as a risk factor for dengue hemorrhagic fever. Am J Trop Med Hyg.

[43] Guzman MG, Kouri G, Valdes L, Bravo J, Vazquez S, Halstead SB (2002). Enhanced severity of secondary dengue-2 infections: Death rates in 1981 and 1997 Cuban outbreaks. Rev Panam Salud Publica.

[44] Guzman MG, Kouri G (2008). Dengue haemorrhagic fever integral hypothesis: Confirming observations, 1987–2007. Trans R Soc Trop Med Hyg.

[45] Alvarez M, Rodriguez-Roche R, Bernardo L, Vazquez S, Morier L, Gonzalez D, Castro O, Kouri G, Halstead SB, Guzman MG (2006). Dengue Hemorrhagic Fever Caused by Sequential Dengue 1–3 Virus Infections over a Long Time Interval: Havana Epidemic, 2001–2002. Am J Trop Med Hyg.

[46] Gollins SW, Porterfield JS (1984). Flavivirus infection enhancement in macrophages: Radioactive and biological studies on the effect of antibody on viral fate. J Gen Virol.

[47] Gollins SW, Porterfield JS (1985). Flavivirus infection enhancement in macrophages: An electron microscopic study of viral cellular entry. J Gen Virol.

[48] Halstead SB, Pasvol G, Hoffman SL (2008). Pathophysiology. Dengue.

[49] Stephenson JR (2005). Understanding dengue pathogenesis: Implications for vaccine design. Bull World Health Organ.

[50] Vaughn DW, Green S, Kalayanarooj S, Innis BL, Nimmannitya S, Suntayakorn S, Endy TP, Raengsakulrach B, Rothman AL, Ennis FA (2000). Dengue viremia titer, antibody response pattern, and virus serotype correlate with disease severity. J Infect Dis.

[51] Libraty DH, Young PR, Pickering D, Endy TP, Kalayanarooj S, Green S, Vaughn DW, Nisalak A, Ennis FA, Rothman AL (2002). High circulating levels of the dengue virus nonstructural protein NS1 early in dengue illness correlate with the development of dengue hemorrhagic fever. J Infect Dis.

[52] Wang WK, Chao DY, Kao CL, Wu HC, Liu YC, Li CM, Lin SC, Ho ST, Huang JH, King CC (2003). High levels of plasma dengue viral load during defervescence in patients with dengue hemorrhagic fever: Implications for pathogenesis. Virology.

[53] Cameron B, Galbraith S, Zhang Y, Davenport T, Vollmer-Conna U, Wakefield D, Hickie I, Dunsmuir W, Whistler T, Vernon S (2007). Gene expression correlates of postinfective fatigue syndrome after infectious mononucleosis. J Infect Dis.

[54] Lidbury BA, Mahalingam S (2000). Specific ablation of antiviral gene expression in macrophages by antibody-dependent enhancement of Ross River virus infection. J Virol.

[55] Chareonsirisuthigul T, Kalayanarooj S, Ubol S (2007). Dengue virus (DENV) antibody-dependent enhancement of infection upregulates the production of anti-inflammatory cytokines, but suppresses anti-DENV free radical and pro-inflammatory cytokine production, in THP-1 cells. J Gen Virol.

[56] Henchal EA, McCown JM, Burke DS, Seguin MC, Brandt WE (1985). Epitopic analysis of antigenic determinants on the surface of dengue-2 virions using monoclonal antibodies. Am J Trop Med Hyg.

[57] Pierson TC, Diamond MS (2008). Molecular mechanisms of antibody-mediated neutralisation of flavivirus infection. Expert Rev Mol Med.

[58] King NJ, Getts DR, Getts MT, Rana S, Shrestha B, Kesson AM (2007). Immunopathology of flavivirus infections. Immunol Cell Biol.

[59] Kaufman BM, Summers PL, Dubois DR, Cohen WH, Gentry MK, Timchak RL, Burke DS, Eckels KH (1989). Monoclonal antibodies for dengue virus prM glycoprotein protect mice against lethal dengue infection. Am J Trop Med Hyg.

[60] Pierson TC (2007). Modeling Antibody-Enhanced Dengue Virus Infection and Disease in Mice: Protection or Pathogenesis. Cell Host Microbe.

[61] Roehrig JT, Mathews JH, Trent DW (1983). Identification of epitopes on the E glycoprotein of Saint Louis encephalitis virus using monoclonal antibodies. Virology.

[62] Throsby M, Geuijen C, Goudsmit J, Bakker AQ, Korimbocus J, Kramer RA, Clijsters-van der Horst M, de Jong M, Jongeneelen M, Thijsse S (2006). Isolation and characterization of human monoclonal antibodies from individuals infected with West Nile Virus. J Virol.

[63] Lok SM, Kostyuchenko V, Nybakken GE, Holdaway HA, Battisti AJ, Sukupolvi-Petty S, Sedlak D, Fremont DH, Chipman PR, Roehrig JT (2008). Binding of a neutralizing antibody to dengue virus alters the arrangement of surface glycoproteins. Nat Struct Mol Biol.

[64] Schieffelin JS, Costin JM, Nicholson CO, Orgeron NM, Fontaine KA, Isern S, Michael SF, Robinson JE (2010). Neutralizing and non-neutralizing monoclonal antibodies against dengue virus E protein derived from a naturally infected patient. Virol J.

[65] Wahala WM, Kraus AA, Haymore LB, Accavitti-Loper MA, de Silva AM (2009). Dengue virus neutralization by human immune sera: Role of envelope protein domain III-reactive antibody. Virology.

[66] Wahala WM, Donaldson EF, de Alwis R, Accavitti-Loper MA, Baric RS, de Silva AM (2010). Natural strain variation and antibody neutralization of dengue serotype 3 viruses. PLoS Pathog.

[67] Blaney JE, Matro JM, Murphy BR, Whitehead SS (2005). Recombinant, live-attenuated tetravalent dengue virus vaccine formulations induce a balanced, broad, and protective neutralizing antibody response against each of the four serotypes in rhesus monkeys. J Virol.

[68] Alvarez M, Pavon-Oro A, Rodriguez-Roche R, Bernardo L, Morier L, Sanchez L, Alvarez AM, Guzman MG (2008). Neutralizing antibody response variation against dengue 3 strains. J Med Virol.

[69] Bray M, Lai CJ (1991). Dengue virus premembrane and membrane proteins elicit a protective immune response. Virology.

[70] Falconar AK (1999). Identification of an epitope on the dengue virus membrane (M) protein defined by cross-protective monoclonal antibodies: Design of an improved epitope sequence based on common determinants present in both envelope (E and M) proteins. Arch Virol.

[71] Vazquez S, Guzman MG, Guillen G, Chinea G, Perez AB, Pupo M, Rodriguez R, Reyes O, Garay HE, Delgado I (2002). Immune response to synthetic peptides of dengue prM protein. Vaccine.

[72] Randolph VB, Winkler G, Stollar V (1990). Acidotropic-amines inhibit proteolytic processing of flavivirus prM protein. Virology.

[73] Huang KJ, Yang YC, Lin YS, Huang JH, Liu HS, Yeh TM, Chen SH, Liu CC, Lei HY (2006). The dual-specific binding of dengue virus and target cells for the antibody-dependent enhancement of dengue virus infection. J Immunol.

[74] Yu IM, Zhang W, Holdaway HA, Li L, Kostyuchenko VA, Chipman PR, Kuhn RJ, Rossmann MG, Chen J (2008). Structure of the immature dengue virus at low pH primes proteolytic maturation. Science.

[75] Rodenhuis-Zybert IA, van der Schaar HM, da Silva Voorham JM, van der Ende-Metselaar H, Lei HY, Wilschut J, Smit JM (2010). Immature dengue virus: A veiled pathogen. PLoS Pathog.

[76] Keelapang P, Sriburi R, Supasa S, Panyadee N, Songjaeng A, Jairungsri A, Puttikhunt C, Kasinrerk W, Malasit P, Sittisombut N (2004). Alterations of pr-M cleavage and virus export in pr-M junction chimeric dengue viruses. J Virol.

[77] Behrens SE, Isken O, Kalitzky M, Borowski P (2006). Cis-and Trans-acting Determinants of Flaviviridae Replication. Molecular Biology of the Flavivirus.

[78] Lai MM (1998). Cellular factors in the transcription and replication of RNA genomesÑ a parallet to DNA-dependent RNA transcription. Virology.

[79] Valdes K, Alvarez M, Pupo M, Vazquez S, Rodriguez R, Guzman MG (2000). Human Dengue antibodies against structural and nonstructural proteins. Clin Diagn Lab Immunol.

[80] Churdboonchart V, Bhamarapravati N, Peampramprecha S, Sirinavin S (1991). Antibodies against dengue viral proteins in primary and secondary dengue hemorrhagic fever. Am J Trop Med Hyg.

[81] Se-Thoe SY, Ng MM, Ling AE (1999). Retrospective study of Western blot profiles in immune sera of natural dengue virus infections. J Med Virol.

[82] Cherrier MV, Kaufmann B, Nybakken GE, Lok SM, Warren JT, Chen BR, Nelson CA, Kostyuchenko VA, Holdaway HA, Chipman PR (2009). Structural basis for the preferential recognition of immature flaviviruses by a fusion-loop antibody. Embo J.

[83] Kouri GP, Guzman MG, Bravo JR (1987). Why dengue haemorrhagic fever in Cuba? 2. An integral analysis. Trans R Soc Trop Med Hyg.

[84] Rico-Hesse R (2007). Dengue virus evolution and virulence models. Clin Infect Dis.

[85] Halstead SB (2007). Dengue. Lancet.

[86] Guzman MG, Kouri G, Bravo J, Valdes L, Vazquez S, Halstead SB (2002). Effect of age on outcome of secondary dengue 2 infections. Int J Infect Dis.

[87] Halstead SB, Streit TG, Lafontant JG, Putvatana R, Russell K, Sun W, Kanesa-Thasan N, Hayes CG, Watts DM (2001). Haiti: Absence of dengue hemorrhagic fever despite hyperendemic dengue virus transmission. Am J Trop Med Hyg.

[88] Bravo JR, Guzman MG, Kouri GP (1987). Why dengue haemorrhagic fever in Cuba? 1. Individual risk factors for dengue haemorrhagic fever/dengue shock syndrome (DHF/DSS). Trans R Soc Trop Med Hyg.

[89] Sierra B, Alegre R, Perez AB, Garcia G, Sturn-Ramirez K, Obasanjo O, Aguirre E, Alvarez M, Rodriguez-Roche R, Valdes L (2007). HLA-A, -B, -C, and -DRB1 allele frequencies in Cuban individuals with antecedents of dengue 2 disease: Advantages of the Cuban population for HLA studies of dengue virus infection. Hum Immunol.

[90] Garcia G, Sierra B, Perez AB, Aguirre E, Rosado I, Gonzalez N, Izquierdo A, Pupo M, Ruiz A, Diaz D (2010). Asymptomatic dengue infection in a Cuban population confirms the protective role of the RR variant of the FcyRIIa polymorphism. Am J Trop Med Hyg.

[91] Sierra B, Kouri G, Guzman MG (2007). Race: A risk factor for dengue hemorrhagic fever. Arch Virol.

[92] Halstead SB, Pasvol G, Hoffman SL (2008). Overview and History. Dengue.

[93] Yamanaka A, Kosugi S, Konishi E (2008). Infection-enhancing and -neutralizing activities of mouse monoclonal antibodies against dengue type 2 and 4 viruses are controlled by complement levels. J Virol.

[94] Balsitis SJ, Williams KL, Lachica R, Flores D, Kyle JL, Mehlhop E, Johnson S, Diamond MS, Beatty PR, Harris E (2010). Lethal antibody enhancement of dengue disease in mice is prevented by Fc modification. PLoS Pathog.

[95] Mehlhop E, Ansarah-Sobrinho C, Johnson S, Engle M, Fremont DH, Pierson TC, Diamond MS (2007). Complement protein C1q inhibits antibody-dependent enhancement of flavivirus infection in an IgG subclass-specific manner. Cell Host Microbe.

[96] Boonnak K, Slike BM, Burgess TH, Mason RM, Wu SJ, Sun P, Porter K, Rudiman IF, Yuwono D, Puthavathana P (2008). Role of dendritic cells in antibody-dependent enhancement of dengue virus infection. J Virol.

